# Characterization of Fitzroy River Virus and Serologic Evidence of Human and Animal Infection

**DOI:** 10.3201/eid2308.161440

**Published:** 2017-08

**Authors:** Cheryl A. Johansen, Simon H. Williams, Lorna F. Melville, Jay Nicholson, Roy A. Hall, Helle Bielefeldt-Ohmann, Natalie A. Prow, Glenys R. Chidlow, Shani Wong, Rohini Sinha, David T. Williams, W. Ian Lipkin, David W. Smith

**Affiliations:** The University of Western Australia, Nedlands, Western Australia, Australia (C.A. Johansen, J. Nicholson, S. Wong, D.W. Smith);; PathWest Laboratory Medicine Western Australia, Nedlands (C.A. Johansen, G.R. Chidlow, D.W. Smith);; Columbia University, New York, New York, USA (S.H. Williams, R. Sinha, W.I. Lipkin);; The Northern Territory Government, Darwin, Northern Territory, Australia (L.F. Melville);; The University of Queensland, St. Lucia, Queensland, Australia (R.A. Hall, H. Bielefeldt-Ohmann, N.A. Prow);; The University of Queensland, Gatton, Queensland, Australia (H. Bielefeldt-Ohmann);; CSIRO Australian Animal Health Laboratory, Geelong, Victoria, Australia (D.T. Williams)

**Keywords:** novel flavivirus, Fitzroy River virus, arbovirus, *Aedes normanensis*, yellow fever virus group, whole-genome sequencing, viruses, Australia, United States

## Abstract

In northern Western Australia in 2011 and 2012, surveillance detected a novel arbovirus in mosquitoes. Genetic and phenotypic analyses confirmed that the new flavivirus, named Fitzroy River virus, is related to Sepik virus and Wesselsbron virus, in the yellow fever virus group. Most (81%) isolates came from *Aedes normanensis* mosquitoes, providing circumstantial evidence of the probable vector. In cell culture, Fitzroy River virus replicated in mosquito (C6/36), mammalian (Vero, PSEK, and BSR), and avian (DF-1) cells. It also infected intraperitoneally inoculated weanling mice and caused mild clinical disease in 3 intracranially inoculated mice. Specific neutralizing antibodies were detected in sentinel horses (12.6%), cattle (6.6%), and chickens (0.5%) in the Northern Territory of Australia and in a subset of humans (0.8%) from northern Western Australia.

In the state of Western Australia, Australia, active surveillance is conducted for mosquitoborne viruses of major human health significance: alphaviruses Ross River virus (RRV) and Barmah Forest virus (BFV) and flaviviruses Murray Valley encephalitis virus (MVEV) and West Nile virus (subtype Kunjin virus; KUNV). These flaviviruses are endemic and epidemic to the northern and central areas of Australia, where surveillance involves year-round testing for seroconversions in sentinel chickens (*1*) and virus isolation from mosquito pools collected annually (*2*,*3*). More frequent mosquito collection is prevented by the logistical difficulties of accessing remote areas. Commonly isolated arboviruses include the flaviviruses MVEV (and subtype Alfuy virus), KUNV, Kokobera virus (KOKV), and Edge Hill virus (EHV) and the alphaviruses RRV, BFV, and Sindbis virus (*4*,*5*). This system occasionally detects viruses that cannot be identified as known viruses, such as Stretch Lagoon virus, an orbivirus isolated in 2002 (*6*). We describe the detection and characterization of a novel flavivirus named Fitzroy River virus (FRV), isolated from mosquitoes collected in northern Western Australia, and seroepidemiologic evidence of human or animal infection.

## Methods

### Adult Mosquito Collections

**Figure 1:**
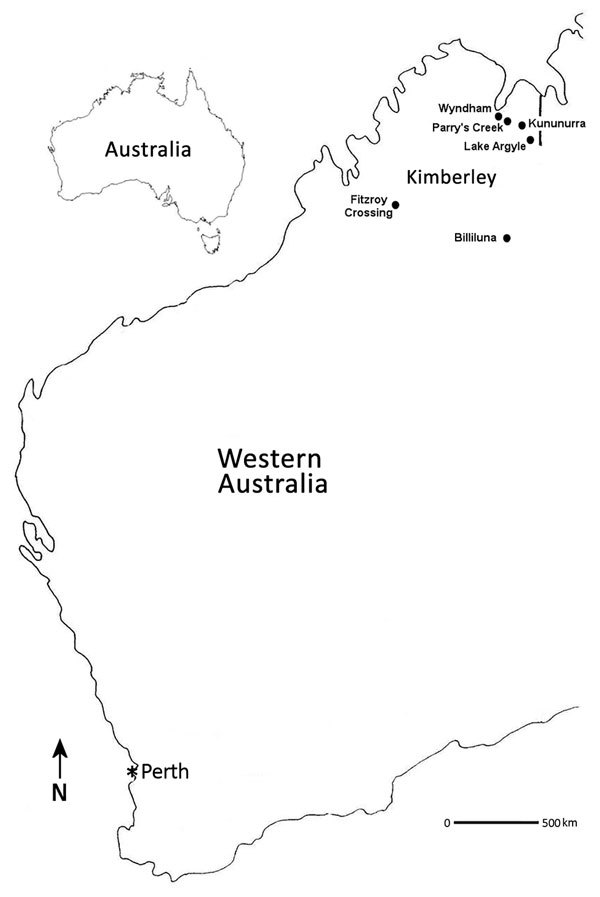
Locations where Fitzroy River virus–positive mosquitoes were collected (black dots), Western Australia, Australia, 2011 and 2012. Perth (asterisk), the capital city and most densely populated area of Western Australia, is shown to indicate its distance from the Kimberley region.

Adult mosquitoes were collected during March and April 2010–2015, at the end of the summer wet season across the Kimberley region of Australia (*2*) (Figure 1). Mosquitoes were collected in encephalitis vector surveillance traps (*7*) baited with carbon dioxide and were separated by species and pooled (*8*–*10*); blood-fed mosquitoes were excluded from analysis.

### Virus Isolation and Identification

Virus isolation from all mosquito pools was performed as previously described (*10*). In brief, mosquito pools were homogenized and serially passaged from C6/36 (*Aedes albopictus* mosquito) cells onto Vero (African green monkey kidney) and PSEK (porcine squamous equine kidney) cells. PSEK cells were later replaced by BSR (baby hamster kidney) cells. Viruses were detected and identified by use of microscopy and monoclonal antibody (mAb) binding patterns in ELISA. For flavivirus-reactive samples, a flavivirus group–reactive 1-step reverse transcription PCR assay (Invitrogen, Carlsbad, CA, USA) (*11*) was used to amplify a 0.6-kb fragment of the nonstructural protein 5 (NS5) and 3′ untranslated region (3′ UTR) for PCR and sequence confirmation.

### Whole-Genome Sequencing

RNA from viral stock (the prototype isolate K73884) was extracted with TRI Reagent (Molecular Research Center, Inc., Cincinnati, OH, USA) and sequenced on a HiSeq ultra high–throughput sequencing platform (Illumina, San Diego, CA, USA). Trimmed reads were assessed for quality by using PRINSEQ version 0.20.2 (*12*) before host (metazoan and mosquito) genome subtraction (Bowtie 2) (*13*) and assembly (MIRA version 4.0) (*14*). Resulting contiguous sequences and unique singletons were subjected to homology search by using MegaBLAST and blastx (https://blast.ncbi.nlm.nih.gov/Blast.cgi) against the GenBank database. Sequences that were similar to viruses from the yellow fever virus (YFV) group, a monophyletic branch that previously included 3 viruses (Wesselsbron virus [WESSV], Sepik virus [SEPV], and YFV) (*15*) were manually edited and reassembled by using Geneious version 7.1.5 (*16*). The complete genome was resequenced by using overlapping PCR and confirmed by bidirectional Sanger sequencing. The sequences for the 5′ and 3′ UTRs were acquired by using the SMARTer RACE cDNA amplification kit (Takara Bio USA, Mountain View, CA, USA).

### Phylogenetic and Recombination Analyses

Nucleotide sequences for the complete polyproteins representing 44 mosquitoborne and tickborne flaviviruses, as well as those that are insect specific or have no known vector, were retrieved from GenBank. Alignments with FRV were performed by using MUSCLE in Geneious version 7.1.5, and a maximum-likelihood phylogenetic tree was constructed by using the general time reversible plus gamma distribution site model of nucleotide substitution with 500 bootstrap replicates (MEGA version 7.0.16) (*17*). Tamana bat virus was used as the outgroup. For alignments of cleavage recognition sequences from members of the YFV group, previously established sites were identified and aligned (*18*,*19*). To assess whether FRV was a recombinant virus, we analyzed polyprotein sequence alignments (as described above) by using default parameters for RDP, GENECONV, BootScan, MaxChi, Chimaera, SisScan, 3SEQ, and Phylpro methods available in the RDP4 program suite (*20*).

### Virus Growth Kinetics in Vitro

Virus replication was assessed in mosquito (C6/36), mammalian (Vero and BSR), and avian (DF-1) cells (*21*) by using a multiplicity of infection of 0.1 in 2% fetal bovine serum in M199 (C6/36 cells) or DMEM (Vero, BSR, and DF-1 cells). After 1 hour of incubation at 28°C (C6/36 cells) or 37°C (Vero, BSR, and DF-1 cells), the inoculum was removed and monolayers were washed before addition of 1 mL of media. Plates were incubated; monolayers examined for cytopathic effect (CPE); and samples removed in triplicate at 0, 1, 2, 3, 4, and 7 days postinoculation (dpi) and stored at −80°C. The 50% tissue culture infectious dose in each sample was determined by serial dilutions and titration in BSR cells in 96-well tissue culture plates, and titers were calculated (*22*).

### Determination of Virulence in Mice

All procedures using animals were approved by The University of Queensland Animal Ethics Committee. Groups of 10 mice (18–19 day weanling CD1 mice, equal numbers of each sex) were challenged by intraperitoneal injection of 50 μL or intracranial injection of 20 μL of either 100 or 1,000 50% tissue culture dose infectious units (IU) of FRV. Groups of 3 mice were mock challenged intraperitoneally or intracranially. Veterinarians monitored the mice twice daily for 19 days and then daily through 21 dpi (*23*,*24*). At 21 dpi, all mice were deeply anesthetized, bled by cardiac puncture, and killed by cervical dislocation. No animals required euthanasia during the experiment. FRV-specific antibodies were detected by fixed-cell ELISA (*25*), and the brains of mice with mild clinical signs were fixed in 10% neutral-buffered formaldehyde and processed for histopathologic and immunohistochemical examination (*23*,*24*).

### Serologic Surveys

Human serologic studies were performed with approval from The University of Western Australia Human Ethics Committee. We used human serum samples that were submitted to PathWest Laboratory Medicine WA (Nedlands, Western Australia, Australia) for arbovirus serologic testing and that were positive by flavivirus hemagglutination inhibition assay. We also used serum samples that were submitted for alphavirus testing only (RRV and BFV) and that were serologically negative. Deidentified information about patient age, sex, ZIP code, and test results were provided. Samples from residents of regions in northern Western Australia where FRV had been detected in mosquitoes or where *Aedes normanensis* mosquitoes are abundant were targeted for this survey. Samples were tested in a flavivirus epitope blocking ELISA that used mAb 3H6 (*26*). Serum containing flavivirus antibodies in ELISA were subsequently tested by serum cross-neutralization assay for antibodies to FRV, MVEV, KUNV, Alfuy virus, KOKV, Stratford virus, and EHV as described previously (*27*). As controls, we used polyclonal rabbit or mouse serum previously raised to these viruses.

Animal serologic studies were approved by The Charles Darwin University Animal Ethics Committee. Animal serum (sentinel cattle, horses, chickens, and wallabies) from the Northern Territory of Australia was tested for antibodies to FRV by neutralization tests (*28*) without prior testing by flavivirus epitope blocking ELISA. Cross-neutralizations with SEPV were conducted on a subset of positive samples to confirm antibody specificity.

## Results

### Viruses and mAb Binding Patterns

We saw little or no visual evidence of infection of C6/36 cell monolayers during the isolation of FRV, and it grew slowly in Vero cells. Cytopathic evidence of infection was most marked in PSEK and BSR cells. Isolates were initially typed by their mAb binding profile against a panel of flavivirus- and alphavirus-reactive mAbs in fixed-cell ELISA. All isolates of FRV reacted with the flavivirus-reactive mAb 4G2 but failed to react with other flavivirus- and alphavirus-reactive mAbs (data not shown). Preliminary analyses of the nucleotide sequence of the NS5–3′ UTR showed 75%–80% identity to SEPV and WESSV, the viruses most closely related to YFV (*15*,*19*,*29*,*30*). Identity between all FRV isolates in the NS5–3′ UTR was 98.9%–100%. The mAb binding profile differed from EHV and SEPV, and positive reactions to FRV were detected with mAbs 4G2 and 4G4 only (Table 1).

**Table 1:** Monoclonal antibody binding pattern of FRV isolates from Western Australia*

Virus	Monoclonal antibody†										
	4G2	4G4	6F7	7C6	8G2	6A9	3D11	3B11	3G1	5D3	7C3
FRV‡	+	+	-	–	–	–	–	–	–	–	–
SEPV	+	+	+	–	–	–	–	–	–	–	–
YFV	+	+	–	–	–	–	–	–	–	–	–
EHV	+	–	+	+	+	+	+	+	+	+	+

*EHV, Edge Hill virus; FRV, Fitzroy River virus; SEPV, Sepik virus; YFV, yellow fever virus; +, positive (optical density of >0.2 and at least 2 times the mean of negative control wells); –, negative.  †Original descriptions of monoclonal antibody from (*31*) (4G2), (*32*) (4G4), (*33*) (6F7), and (*34*) (7C6, 8G2, 6A9, 3D11, 3B11, 3G1, 5D3, and 7C3). ‡Monoclonal antibody binding patterns of all FRV isolates were identical to those of the prototype isolate K73884.

### Whole-Genome Sequences and Phylogeny

Unbiased high-throughput sequencing results provided >99% of the FRV genome with only partial UTRs not obtained. The completed full-genome length of FRV was 10,807 nt with a single 10,218-nt open reading frame flanked by a 117-nt 5′ UTR and a 472-nt 3′ UTR (Table 2; Technical Appendix Figure, panel A) and has been deposited in GenBank under accession no. KM 361634. At the amino acid level, FRV was more similar to SEPV than to WESSV in most regions, with the exception of the short 2k peptide (70% vs. 91% aa homology), NS4a (87% vs. 92% aa homology), and NS5 (88% vs. 89% aa homology). Because there was a sharp change in amino acid homology between FRV and SEPV across the 2k peptide (70%) and NS4b (94%), we assessed aligned polyprotein sequences for recombination breakpoints by using 8 algorithms in the RDP4 suite, but we found no evidence suggesting that recombination had occurred (data not shown).

**Table 2:** Comparison of genomic region lengths and similarities between members of the YFV group and FRV*

Genomic region	Virus

FRV			SEPV			WESSV			YFV	
nt	aa	nt	aa	nt	aa	nt	aa
5′ UTR	117	NA		116 (94)	NA		118 (92)	NA		118 (52)	NA
Capsid	348	116		348 (77)	116 (84)		348 (74)	116 (78)		363 (54)	121 (39)
Premembrane	267	89		267 (78)	89 (94)		267 (73)	89 (87)		267 (59)	89 (59)
Membrane	225	75		225 (80)	75 (96)		225 (74)	75 (87)		225 (58)	75 (47)
Envelope	1,470	490		1,470 (81)	490 (96)		1,470 (79)	490 (93)		1,479 (63)	493 (54)
NS1	1,059	353		1,059 (79)	353 (93)		1,059 (76)	353 (87)		1,056 (63)	352 (64)
NS2a	678	226		678 (78)	226 (88)		678 (77)	226 (85)		672 (54)	224 (40)
NS2b	390	130		390 (78)	130 (88)		390 (76)	130 (88)		390 (56)	130 (50)
NS3	1,869	623		1,869 (79)	623 (93)		1,869 (77)	623 (92)		1,869 (67)	623 (71)
NS4a	378	126		378 (75))	126 (87)		378 (75)	126 (92)		378 (61)	126 (57)
2k	69	23		69 (71)	23 (70)		69 (74)	23 (91)		69 (61)	23 (57)
NS4b	744	248		744 (79)	248 (94)		744 (77)	248 (91)		750 (66)	250 (64)
NS5	2,721	906		2,721 (78)	906 (88)		2,721 (76)	906 (89)		2,718 (66)	905 (68)
3′ UTR	472	NA		459 (86)	NA		478 (84)	NA		508 (65)	NA
Polyprotein	10,218	3,405		10,218 (79)	3,405 (91)		10,218 (77)	3,405 (89)		10,236 (63)	3,411 (61
Full genome	10,807	NA		10,793 (79)	NA		10,814 (77)	NA		10,862 (62)	NA

*Values are sequence length (% identity with FRV). FRV, Fitzroy River virus (GenBank accession no. KM 361634); NS, nonstructural; SEPV, Sepik virus (GenBank accession no. NC008719); UTR, untranslated region; WESSV, Wesselsbron virus (accession no. JN226796); YFV, yellow fever virus (X03700); NA, not applicable.

FRV was highly similar to SEPV across the structural viral proteins, including the membrane (96%) and envelope (96%) proteins. Over the full genome, FRV displayed the highest nucleotide identity to SEPV (79%), WESSV (77%), and YFV (62%). These lower nucleotide identities are in contrast to the polyprotein amino acid homologies (SEPV 91%, WESSV 89%, YFV 61%), indicating that a large proportion of nucleotide differences between FRV and SEPV or WESSV were synonymous. The level of nucleotide identity was higher in the 5′ and 3′ UTRs (94% and 86%, respectively) than in structural proteins (up to 81%) for FRV and SEPV, reflecting the functional importance of the UTRs for virus replication. Closer analysis of several conserved features of flavivirus UTRs is shown in the Technical Appendix Figure. Although comparisons of the cyclization sequences (Technical Appendix Figure, panel C) indicate a high level of conservation among all members of the YFV group, alignments of the upstream AUG region (Technical Appendix Figure, panel D) highlight a clear separation of FRV, SEPV, and WESSV from YFV. When we assessed the string of tandem repeats in the 3′ UTR, some differences between FRV, SEPV, and WESSV emerged. We identified 3 highly conserved repeats in the 3′ UTR of FRV (RFR1, RFR2, and RFR3; Technical Appendix Figure, panel B), which are most similar to the previously described sequences identified in YFV (RYF1, RYF2, and RYF3). Strikingly, WESSV (69.7%–90.6%) and FRV (72.7%–90.6%) retained homologous sequences to RYF1, RYF2, and RYF3, and SEPV retained only a vestigial repeat sequence (RSEP3) that is highly divergent from all other members of the YFV group, including FRV.

**Figure 2:**
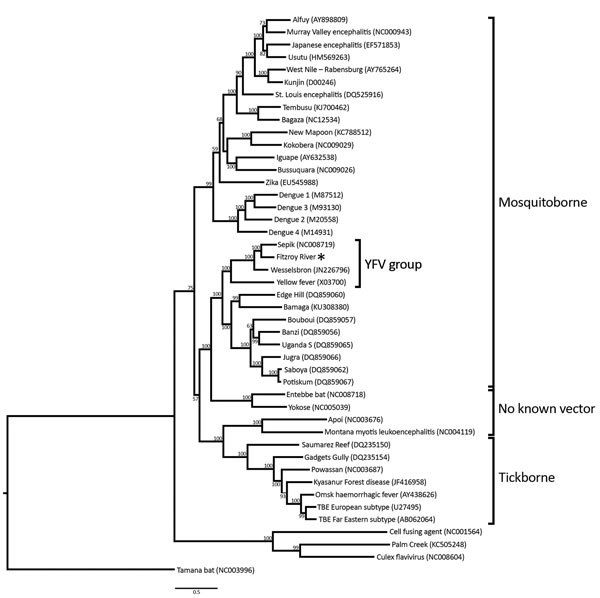
Phylogenetic tree of the genus *Flavivirus*, based on full polyprotein nucleotide sequences. Asterisk (*) indicates Fitzroy River virus. Scale bar indicates nucleotide substitutions per site. YFV, yellow fever virus.

FRV shares a common ancestor with SEPV (with 100% bootstrap support) (Figure 2) and is located in the distinct YFV group according to International Committee on Taxonomy of Viruses classification (*15*). Analysis of the 12 cleavage sites located within the polyprotein open reading frame, following the scheme described by Kuno and Chang (*19*), supported the phylogenetic structure of the YFV group; YFV displayed marked divergence from FRV, SEPV, and WESSV in several cleavage sites, including Ci/PrM, NS1/NS2a, NS2a/NS2b, and NS2b/NS3 (Technical Appendix Figure, panel E).

### Viruses Isolated from Mosquitoes

Mosquitoes yielding isolates of FRV were collected at Fitzroy Crossing in the West Kimberley region in 2011 (Table 3); FRV was isolated from 2 pools of *Ae. normanensis* and 1 pool of *Anopheles amictus* mosquitoes. In 2012, a similar level of sampling in the same geographic area (data not shown) showed a shift of activity away from Fitzroy Crossing to a broader area in the eastern and southern Kimberley region, encompassing Billiluna, Kununurra, and Wyndham (Table 3). Sixteen isolates were obtained, most (81.2%) from *Ae. normanensis* mosquitoes (Table 3) and all from female mosquitoes. Additional virus was isolated from *An. amictus*, *Culex annulirostris*, and a pool of damaged and unidentifiable *Aedes* spp. mosquitoes. The minimum infection rate was greatest at Billiluna (2.5 FRV-infected mosquitoes/1,000 mosquitoes; Table 4). Other arboviruses detected during these seasons included MVEV, KUNV, KOKV, RRV, and Sindbis virus (Table 3).

**Table 3:** Mosquito species collected and arboviruses isolated from the Kimberley region of Western Australia, Australia, 2011 and 2012*

Year, location, mosquito species	No. (%) collected	No. processed	No. pools processed	No. virus isolates
2011				
Fitzroy Crossing				
* Ae. (Ochlerotatus) normanensis*	4,657 (38.4)	2,497	110	2 FRV, 3 non A/F
* An. (Cellia) amictus*	750 (6.2)	504	29	1 FRV, 4 non A/F
*An. (Cellia) annulipes* s.l.	2,879 (23.7)	1,898	84	6 non A/F
* Cx. (Culex) annulirostris*	3,202 (26.4)	1,773	79	2 MVEV, 1 KUNV, 1 KUNV+SINV, 6 non A/F
Other	635 (5.2)	482	100	1 non A/F†
Subtotal	12,123 (100)	7,154	402	
2012				
Billiluna				
* Ae. (Macleaya) tremulus*	252 (2.0)	135	14	
* Ae. (Ochlerotatus) normanensis*	1,679 (13.4)	1,244	58	3 FRV
* An. (Cellia) amictus*	650 (5.2)	508	42	
*An. (Cellia) annulipes* s.l.	3,456 (27.5)	1,555	74	
* An. (Cellia) novaguinensis*	247 (2.0)	152	18	
* Cx. (Culex) annulirostris*	5,608 (44.6)	3,424	148	2 MVEV
Damaged *Anopheles* spp.	131 (1.0)	83	14	
Damaged *Culex* spp.	218 (1.7)	111	15	
Other	326 (2.6)	199	84	
Subtotal	12,567 (100)	7,411	467	
Kununurra				
* Ae. (Finlaya) notoscriptus*	455 (1.3)	381	31	
* Ae. (Neomellanoconion) lineatopennis*	3,457 (9.9)	1540	80	
* Ae. (Ochlerotatus) normanensis*	12,632 (36.2)	4917	219	7 FRV, 2 RRV
* An. (Anopheles) bancroftii*	2,428 (7.0)	723	52	
*An. (Cellia) annulipes* s.l.	1,758 (5.0)	1025	67	
* An. (Cellia) meraukensis*	2,717 (7.8)	863	60	
* Cq. (Coquillettidia) xanthogaster*	931 (2.7)	796	55	
* Cx. (Culex) annulirostris*	7,600 (21.8)	4195	193	1 RRV
* Ve. (Verrallina) reesi*	468 (1.3)	287	33	
Damaged *Culex* spp.	350 (1.0)	253	30	1 RRV
Other	2,111 (6.0)	1528	369	4 RRV‡
Subtotal	34,907 (100)	16508	1189	
Wyndham				
* Ae. (Ochlerotatus) normanensis*	1,661 (5.4)	551	30	1 FRV, 1 RRV
* An. (Anopheles) bancroftii*	532 (1.7)	122	14	
* An. (Cellia) amictus*	1,589 (5.1)	380	26	
*An. (Cellia) annulipes* s.l.	982 (3.2)	262	20	
* An. (Cellia) meraukensis*	1,677 (5.4)	450	27	
* Cx. (Culex) annulirostris*	21,388 (69.1)	5,357	224	1 FRV, 2 KOKV, 4 RRV
* Cx. (Culex) crinicauda*	330 (1.1)	62	14	
Damaged *Culex* spp.	907 (2.9)	247	17	1 RRV
Other	1,881 (6.1)	898	206	1 FRV, 1 RRV§
Subtotal	30,947 (100)	8329	578	
Total	90,544	39,402	2,636	

*Only mosquito collection locations that yielded isolates of FRV are shown; species collected at abundance of <1.0% are grouped as “other”; named species are female mosquitoes only. Results from male mosquitoes are included in “other.” *Ae., Aedes*; *An., Anopheles*; *Cq*., *Coquillettidia*; *Cx., Culex*; FRV, Fitzroy River virus; KOKV, Kokobera virus; KUNV, West Nile (Kunjin) virus; non A/F, not an alphavirus or flavivirus and identity is yet to be determined, MVEV, Murray Valley encephalitis virus; RRV, Ross River virus; SINV, Sindbis virus; *Ve.,* *Verrallina*.  †Isolated from female *Ae. lineatopennis* mosquito. ‡Isolated from female *Aedeomyia* *catasticta* (1), *Anopheles amictus* (1), and *Mansonia uniformis* (2) mosquitoes. §FRV isolated from a pool of damaged female *Aedes* spp. mosquitoes, RRV isolated from female *An. bancroftii* mosquitoes.

**Table 4:** Minimum infection rates of mosquitoes infected with FRV, Western Australia, Australia, 2011 and 2012*

Year, location, mosquito species	No. isolates	Minimum infection rate†
2011		
Fitzroy Crossing		
* Ae. (Ochlerotatus) normanensis*	2	0.8
* An. (Cellia) amictus*	1	2.0
2012		
Billiluna		
* Ae. (Ochlerotatus) normanensis*	3	2.5
Kununurra		
* Ae. (Ochlerotatus) normanensis*	7	1.4
Wyndham		
* Ae. (Ochlerotatus) normanensis*	1	1.8
* Cx. (Culex) annulirostris*	1	0.2
Damaged *Aedes* spp.	1	1.1

*Ae., *Aedes*; *An*., *Anopheles*; *Cx*, *Cule*x; FRV, Fitzroy River virus. †No. FRV-infected mosquitoes/1,000 mosquitoes; calculated according to (*35*).

### In Vitro Virus Replication

FRV replicated in all 4 cell lines tested. At all time points, the FRV titer grew higher in BSR than in other cell lines, with the exception of Vero cells on day 7; the difference was usually significant (Table 5). Mild CPE was not apparent until day 4 in BSR cells and day 7 in Vero and DF-1 cells; no CPE was evident in C6/36 cells.

**Table 5:** Fitzroy River virus replication in 4 cell lines

Day	Mean Fitzroy River virus titer*			
	C6/36	Vero	BSR	DF-1
1	0^a^	0^a^	3.07 ± 0.06^b^	0^a^
2	4.68 ± 0.21^a^	3.78 ± 0.05^b^	5.13 ± 0.06^c^	3.81 ± 0.3^ab^
3	5.58 ± 0.05^a^	4.72 ± 0.02^b^	6.67 ± 0.08^c^	5.5 ± 0.08^a^
4	6.66 ± 0.09^a^	5.02 ± 0.07^b^	6.72 ± 0.05^a^	6.26 ± 0.15^a^
7	4.41 ± 0.14^a^	7.01 ± 0.01^b^	6.6 ± 0.21^b^	4.25 ± 0.23^a^

*Statistical significance of log transformed arithmetic means was determined with 2-way analysis of variance with correction for multiple comparisons and using the Tukey method for pairwise multiple comparisons (GraphPad Prism version 6.0; GraphPad Software Inc, San Diego, CA, USA). Means in the same row followed by the same superscript letter did not differ significantly (p>0.05). Results at day 0 were excluded because virus detected at this time point represented residual inoculum.

### Virus Virulence in Mice

Two female mice in the 1,000 IU intracerebrally inoculated group and 1 female mouse in the 100 IU intracerebrally inoculated group had hind limb weakness, intermittent photophobia, and/or mild retrobulbar swelling between 5 and 12–13 dpi; however, only 1 female mouse in each intracerebrally inoculated group received a score of 1 on 1–2 days (days 5 and 9). By 13–14 dpi, all mice appeared to be clinically healthy. The only abnormality in intraperitoneally inoculated mice was mild photophobia in 1 mouse in the 1,000 IU group at 6 dpi. Mock-challenged animals showed no clinical abnormalities throughout the study period. All intracerebrally inoculated mice seroconverted; antibody titers were >160. In the intraperitoneally inoculated group, 6 mice in the 100 IU group seroconverted and 8 mice in the 1,000 IU group seroconverted (titers 40 to >160), indicating successful FRV replication.

**Figure 3:**
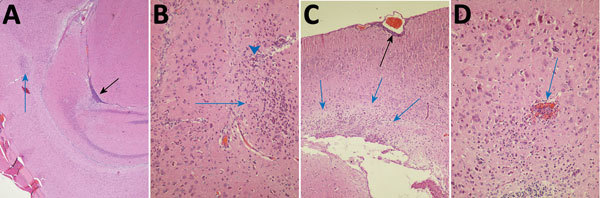
Photomicrographs of Fitzroy River virus (FRV)–induced meningoencephalitis in weanling mice inoculated with 1,000 infectious units of FRV. Panels show multifocal mild to severe perivascular and neuropil infiltration of lymphocytes and monocytes (blue arrows in A–C); meningitis in a sulcus (black arrow in A); glial cell activation with notable astrocytosis, neuron degeneration, and neuronophagia (arrowhead in B); occasional hemorrhage (blue arrow in D); mild periventricular spongiosis (blue arrows in C); and meningitis (black arrow in C). Hematoxylin and eosin staining. Original magnifications: A) ×40, B) ×400, C) ×100, D) ×400.

For the 3 mice in which subtle clinical signs developed, we processed the heads for histopathology. We found histopathologic signs of meningoencephalitis in all 3 (Figure 3, panels A–D). The lesions were most notable in the 1,000 IU intracerebrally inoculated group, corroborating the mild clinical signs observed. The least severe lesions were seen in the mouse from the 100 IU intracerebrally inoculated group; however, the lesions were unilateral in the hemisphere not inoculated. In the other 2 mice, the trend was toward greater severity in the inoculated hemisphere; however, in the other hemisphere and distant from the inoculation site, we found leukocyte infiltration, gliosis, and neuronal degeneration. No viral antigen was detected in the affected brains by immunohistochemistry, suggesting that FRV was cleared at the time of euthanasia (21 dpi).

### Antibodies in Humans and Animals

A total of 366 serum samples from humans from northern Western Australia, submitted to PathWest Laboratory Medicine WA for alphavirus or flavivirus serologic testing from March through May in 2014 and 2015, were tested for antibodies to FRV (Technical Appendix Table). Overall, the prevalence of antibodies to flaviviruses in the ELISA was 33.6%, of which initial screening showed an FRV neutralization titer >10 in 9 samples. For 3 of these samples, cross-neutralization titers showed FRV antibody titers >40 and at least a 4-fold difference between antibody titer to FRV and other flaviviruses from Australia (Table 6), yielding an FRV positivity rate of 0.8% (3/336) of all samples tested and 2.4% (3/123) of samples with evidence of a flavivirus infection by ELISA. All 3 FRV antibody–positive samples were from the West Kimberley (Broome) region.

**Table 6:** Serologic test results for 9 serum samples from humans from northern Western Australia, which contained FRV neutralizing antibodies at initial testing*

Sample	ELISA, % block	FRV initial neutralization titer	Cross-neutralization titers							Infecting virus
FRV	MVEV	KUNV	ALFV	KOKV	STRV	EHV
2014–1	79	10	<10	<10	<10	<10	<10	<10	<10	UD
2014–2	87	20	<10	10	10	<10	10	10	40	EHV
2014–3	86	40	<10	<10	<10	<10	<10	20	80	EHV
2014–4	84	160	40	<10	10	<10	<10	10	<10	FRV
2015–1	90	80	<10	<10	160	<10	<10	<10	<10	KUNV
2015–2	92	20	<10	<10	80	<10	<10	<10	<10	KUNV
2015–3	98	40	80	<10	10	<10	<10	<10	<10	FRV
2015–4	95	80	80	10	20	<10	10	10	20	FRV
2015–5	98	10	<10	<10	10	<10	<10	<10	<10	UD

*ALFV, Alfuy virus (K74157); EHV, Edge Hill virus (K74003); FRV, Fitzroy River virus (K73884); KOKV, Kokobera virus (K69949); KUNV, West Nile (Kunjin) virus (K81136); MVEV, Murray Valley encephalitis virus (K68150); STRV, Stratford virus (C338); UD, undetermined**.**

Serum from 227 sentinel cattle, 87 horses, and 178 sentinel chickens from the Northern Territory sampled from 2009/10 through 2014/15 were tested for antibodies to FRV. Neutralizing antibodies to FRV were detected in horses (12.6%) and cattle (6.6%). FRV and SEPV cross-neutralization tests on a subsample of FRV antibody–positive serum samples indicated that the FRV infections were not the result of serologic cross-infection with closely related SEPV (data not shown), which occurs in neighboring Papua New Guinea. One sentinel chicken had a low FRV antibody titer. Most FRV infections in domestic animals (n = 24; 77%) were from 2012/13. We also tested serum from 25 wallabies, 1 wallaroo, and 1 bandicoot from the Northern Territory, collected from 2006/07 through 2013/14. Low levels of FRV antibodies were found in 1 wallaby from the Darwin region in January 2007. We found no association between infection and clinical disease in any cattle, horses, chickens, or marsupials tested.

## Discussion

The new flavivirus from northern Australia, for which we proposed the name Fitzroy River virus, was first isolated from *Ae. normanensis* mosquitoes collected near the Fitzroy River. Phylogenetic analysis of isolate K73884 demonstrates that FRV belongs to the YFV group (*15*). Cleavage recognition sequence analysis groups FRV together with WESSV and SEPV, but distinct from YFV, reflecting the pattern of amino acid homology of members of the YFV group across the polyprotein. When homology of individual viral proteins is assessed, FRV is most closely related to SEPV in all regions excluding some nonstructural components that have a required role in replication (*36*,*37*), notably NS4a and 2k, where homology to WESSV was higher. We found no evidence of recombination breakpoints occurring along the FRV genome, and the low (70%) amino acid homology observed in the 2k peptide is most likely the result of a collection of nonsynonymous mutations. However, this recombination analysis was limited to 44 reference flavivirus sequences; a larger collection that includes more YFV group isolates from the Southeast Asia region may reveal further insights into the evolutionary history of FRV.

The phylogenetic placement of FRV in the YFV group is further supported by analysis of features in the flavivirus UTRs (cyclization sequence, upstream AUG region, and tandem repeats), sequences that are functionally necessary for enhancing replication through formation of secondary RNA structures (*38*). Although we observed near complete consensus in the cyclization sequence at each terminus between these 4 viruses, FRV had higher levels of identity to SEPV and WESSV than YFV across the respective upstream AUG regions. Conversely, the tandem repeats found in the 3′ UTR showed consistently high nucleotide identities (>80% across all 3 sites) with YFV rather than WESSV and SEPV. Together, these data indicate that FRV possesses a unique collection of sequence signatures that distinguish it from other members of the YFV group.

The origin of FRV is unknown. Although increased surveillance in neighboring countries is needed, arbovirus and mosquito monitoring has been pursued in northern Western Australia since the early 1970s (*39*,*40*) with only minor changes in strategy. FRV was not detected by virus culture from earlier mosquito collections, a finding consistent with recent introduction into Western Australia and possibly elsewhere in Australia, thus highlighting the value of ongoing surveillance activities. We cannot exclude the possibility that FRV was circulating in mosquitoes of species (or other insect vectors) not commonly collected in traps routinely used for surveillance of adult mosquitoes in Western Australia and that genetic changes enabled the virus to adapt to a new host species, as has been seen with chikungunya virus (*41*).

Phylogenetic analysis indicated that FRV is most closely related to SEPV in the YFV group, which is currently found only in Papua New Guinea, and WESSV, which occurs in Africa and Thailand. Recent experience with introduction of likely or suspected arbovirus and arbovirus vectors into northern Australia suggests that FRV was probably introduced from Southeast Asia. Included are introductions by mosquitoes such as *Aedes aegypti* (L.), *Aedes vexans*, and *Culex gelidus* (*42*,*43*) and introductions of viruses including Japanese encephalitis virus from Papua New Guinea (*44*), bluetongue viruses from Southeast Asia (*45*,*46*), and epizootic hemorrhagic disease virus 1 from Indonesia (*47*).

Most (81%) FRV has been isolated from *Ae. normanensis* mosquitoes, providing circumstantial evidence that this species may be the dominant vector. Mosquito collections at each locality were conducted ≈2–3 weeks after a period of high rainfall following a relatively dry period. These conditions favor an abundance of *Ae. normanensis* mosquitoes because these mosquitoes rapidly hatch from desiccation-resistant eggs (*48*). The detection of antibodies to FRV in sentinel animals from the Northern Territory is consistent with the range and feeding behavior of *Ae. normanensis* mosquitoes and indicates a wide distribution of FRV in northern Australia.

Our finding of serologic evidence of human infection by FRV, despite low prevalence and apparent confinement to the West Kimberley region, is noteworthy. We detected FRV more extensively across northern Western Australia, so further human infections are likely. Because these samples had been sent for routine diagnostic arbovirus testing, it is presumed that most persons had a clinical illness of concern; however, we did not have access to detailed clinical information. Also, because the samples were single rather than paired acute- and convalescent-phase samples, we could not determine whether the FRV antibodies are the result of acute or previous infections. The antibody titers to FRV in humans were low, and although the cross-neutralizations included all known Australian flaviviruses that replicate in the cell lines we used, these persons may have been infected with an unrecognized flavivirus.

The close relationship of FRV with WESSV and SEPV may indicate potential for FRV to affect domestic animals such as cattle, goats, and sheep. Cattle stations are a dominant agricultural feature of northern Australia. Given that most FRV was isolated from *Ae. normanensis* mosquitoes, that mosquitoes of this species readily feed on cattle and horses, and that the FRV antibody prevalence in sentinel cattle and horses in the Northern Territory was high, we believe that the enzootic transmission cycle for FRV probably involves *Ae. normanensis* mosquitoes and domestic animals such as cattle and horses. Infection with FRV was not associated with clinical disease in animals but could potentially be disguised by other arbovirus infections, such as bovine ephemeral fever (*49*).

The finding of mild clinical signs in FRV-infected weanling mice, more often in those that were intracerebrally infected, indicates that severe clinical disease may be unlikely unless the health of the animal host is compromised. Further research is required to determine if FRV causes clinical disease in humans or domestic animals. The outcomes of this study demonstrate the value of surveillance for mosquitoborne viruses in the detection, characterization, and impact assessment of novel and known arboviruses.

Technical Appendix Detection of neutralizing antibodies to Fitzroy River virus in humans and animals in northern Western Australia and the Northern Territory, Australia, and additional details on the characterization of Fitzroy River virus.
