# Supplementary material for: Characterization of Fitzroy River Virus and Serologic Evidence of Human and Animal Infection
Source: Emerg Infect Dis. 2017 Aug;23(8):1289–99. doi: 10.3201/eid2308.161440 (PMC5547785; doi:10.3201/eid2308.161440)
Supplement: Technical Appendix — Detection of neutralizing antibodies to Fitzroy River virus in humans and animals in northern Western Australia and the Northern Territory, Australia, and additional details on the characterization of Fitzroy River virus. [file 16-1440-Techapp-s1.pdf]

Article DOI: https://doi.org/10.3201/eid2308.161440

# Characterization of Fitzroy River Virus and Serologic Evidence of Human and Animal Infection

**Technical Appendix Table.** Detection of neutralizing antibodies to Fitzroy River virus in humans and animals in northern Western Australia and Northern Territory, Australia*

| | Year | | | | | | | | | | | | | |
|---|---|---|---|---|---|---|---|---|---|---|---|---|---|---|
| | 2009/10 | | 2010/11 | | 2011/12 | | 2012/13 | | 2013/14 | | 2014/15 | | Total | |
| Animal, location | No. tested | No. (%) pos | No. tested | No. (%) pos | No. tested | No. (%) pos | No. tested | No. (%) pos | No. tested | No. (%) pos | No. tested | No. (%) pos | No. tested | No. (%) pos |
| Humans, Western Australia | | | | | | | | | | | | | | |
| West Kimberley | | | | | | | | | 150 | 1 (0.7) | 117 | 2 (1.7) | 267 | 3 (1.1) |
| Northeast Kimberley | | | | | | | | | 50 | | 38 | | 88 | |
| Southeast Kimberley | | | | | | | | | 4 | | 7 | | 11 | |
| Subtotal | | | | | | | | | 204 | 1 (0.5) | 162 | 2 (1.2) | 366 | 3 (0.8) |
| Cattle, Northern Territory | | | | | | | | | | | | | | |
| Katherine Research Station† | 21 | 1 (4.8) | 23 | | 23 | | 23 | 6 (26.1) | | | | | 90 | 7 (7.8) |
| Beatrice Hill Farm‡ | | | | | | | 24 | 1 (4.2) | 22 | 1 (4.5) | | | 46 | 2 (4.3) |
| Douglas Daly Research Farm | | | | | | | 17 | | 17 | | | | 34 | |
| Victoria River Research Station | 20 | | 19 | | | | 18 | 6 (33.3) | | | | | 57 | 6 (10.5) |
| Subtotal | 41 | 1 (2.4) | 42 | | 23 | | 82 | 13 (15.9) | 39 | 1 (2.6) | | | 227 | 15 (6.6) |
| Horses, Northern Territory | | | | | | | | | | | | | | |
| Mataranka | 8 | | | | | | | | | | | | 8 | |
| Cave Creek | 3 | | | | | | | | | | | | 3 | |
| Beatrice Hill | | | 4 | | | | | | | | | | 4 | |
| Douglas Daly | | | 4 | | | | | | | | | | 4 | |
| Kirkimbie | | | 5 | | | | | | | | | | 5 | |
| Humbert River | | | | | 8 | | | | | | | | 8 | |
| Katherine | | | | | 4 | | 5 | 3 (60.0) | | | | | 9 | 3 (33.3) |
| Wave Hill | | | | | 10 | | | | | | | | 10 | |
| Alice Springs | | | | | 2 | | | | | | | | 2 | |
| Victoria River | | | | | 10 | | 3 | 2 (66.7) | | | | | 13 | 2 (15.4) |
| Avago | | | | | 3 | | | | | | | | 3 | |
| Darwin | | | | | 3 | | | | | | | | 3 | |
| Killarney | | | | | | | 15 | 6 (40.0) | | | | | 15 | 6 (40.0) |
| Subtotal | 11 | | 13 | | 40 | | 23 | 11 (47.8) | | | | | 87 | 11 (12.6) |
| Chickens, Northern Territory | | | | | | | | | | | | | | |
| Beatrice Hill | | | | | 12 | | | | | | | | 12 | |
| Gove | | | | | 6 | | | | | | | | 6 | |
| Leanyer | | | | | 16 | 1(6.3) | | | | | 7 | | 23 | 1 (4.3) |
| Howard Springs | | | | | 9 | | | | | | 10 | | 19 | |
| Katherine | 12 | | 10 | | 19 | | | | 11 | | | | 52 | |
| Nathan River | 10 | | 12 | | | | 5 | | | | | | 27 | |
| Tennant Creek | 11 | | 11 | | | | 10 | | 7 | | | | 39 | |

| Animal, location | 2009/10 No. tested | 2009/10 No. (%) pos | 2010/11 No. tested | 2010/11 No. (%) pos | 2011/12 No. tested | 2011/12 No. (%) pos | 2012/13 No. tested | 2012/13 No. (%) pos | 2013/14 No. tested | 2013/14 No. (%) pos | 2014/15 No. tested | 2014/15 No. (%) pos | Total No. tested | Total No. (%) pos |
|---|---|---|---|---|---|---|---|---|---|---|---|---|---|---|
| Subtotal | 33 | | 33 | | 62 | 1 (1.6) | 15 | | 18 | | 17 | | 178 | 1 (0.6) |
| Total | 85 | 1 (1.2) | 88 | | 125 | 1 (0.8) | 120 | 24 (20.0) | 261 | 2 (0.8) | 179 | 2 (1.1) | 858 | 30 (3.5) |

*Pos, positive.
†Includes 1 seroconversion from November through January 2012 and 2013 and 1 from March through April 2012 and 2013.
‡Includes 1 seroconversion from March and April 2012 and 2013.

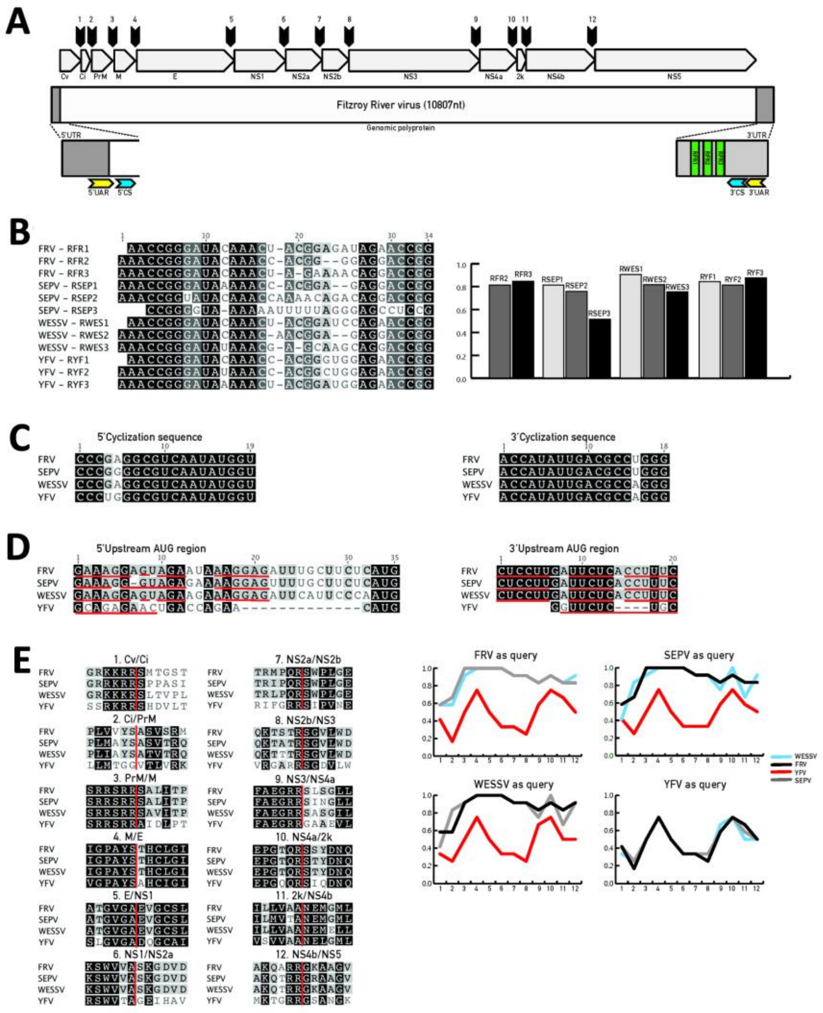


**Technical Appendix Figure**. A) Genome of Fitzroy River virus in 5′→3′ orientation, showing post-translational cleavage sites (filled black arrows) and conserved UTR sequences, CS - cyclization sequence (blue arrow), UAR - upstream AUG region (yellow arrow), RFR - FRV tandem repeat sequence (green box), Cv – viral capsid, Ci – intracellular capsid, PrM – premembrane, M – membrane, E – envelope, NS – non-structural. (B)

Nucleotide alignment of upstream AUG region and graph showing nucleotide distances of tandem repeats 1 to 3 (RFR1–3) for FRV, Sepik virus (SEPV), Wesselsbron virus (WESSV) and yellow fever virus (YFV). (C) Nucleotide alignment of cyclization sequence (D) Nucleotide alignment of upstream AUG region. Red underline highlights the inverted complementary sequence. (E) Amino acid alignment of cleavage recognition sites. The red line denotes cleavage site. Line graphs show percent identity across each cleavage site, numbered.
